# Horizontal and vertical self-paced saccades as a diagnostic marker of traumatic brain injury

**DOI:** 10.2217/cnc-2019-0001

**Published:** 2019-07-25

**Authors:** Melissa Hunfalvay, Claire-Marie Roberts, Nick Murray, Ankur Tyagi, Hannah Kelly, Takumi Bolte

**Affiliations:** 1 RightEye LLC, 7979 Old Georgetown Rd, Suite 801, Bethesda, MD 20814, USA; 2 University of the West of England, Department of Psychology, Bristol, Coldharbour Lane, Bristol, BS16 1QY, UK; 3 East Carolina University, College of Health & Human Performance, Minges Coliseum 166, Greenville, NC 27858, USA; 4 Emory University, Health Sciences, 201 Dowman Dr, Atlanta, GA 30322, USA

**Keywords:** concussion, eye tracking, horizontal saccades, TBI, vertical saccades, VOMS

## Abstract

**Aim:**

Eye tracking tests to measure horizontal and vertical saccades as a proxy for neural deficits associated with traumatic brain injury (TBI) were evaluated in the present study.

**Methodology:**

A total of 287 participants reporting either no TBI, mild, moderate or severe TBI participated in a suite of eye tracking tests to measure horizontal and vertical saccadic performance.

**Results:**

The horizontal saccades test offered a sensitivity of 0.77 and a specificity of 0.78, similarly the vertical saccades tests offered a sensitivity of 0.64 and a specificity of 0.65.

**Conclusion:**

The results indicated that using eye-tracking technology to measure these metrics offers an objective, reliable and quantifiable way of differentiating between individuals with different severities of TBI, and those without a TBI.

The rising incidence of traumatic brain injury (TBI) is an international public health concern and a significant cause of morbidity and mortality [1]. In the USA alone, about 1.7 million people suffer from a TBI every year with 52,000 of these cases resulting in death [2]. According to the CDC, the number of hospitalizations and deaths related to TBI increased by >50% from 2006 to 2014 [3]. Oculomotor research contributes to the growing understanding of TBI by providing insight into neural functioning for clinicians and neuroscientists. Oculomotor behavior is a promising neuropsychological endophenotype, as it reflects abnormalities of complex neurocircuitry [4]; for example, oculomotor impairments have been associated with functional neuroimaging of the brain to examine the effect of neural dysfunction on oculomotor performance [5].

Oculomotor behavior is commonly broken down into the following eye movement categories: fixations, smooth pursuits and saccades [6]. Fixations keep the eye position in a relatively still state to hold the image of a stationary target on the fovea, a site of high visual acuity [7]. Smooth pursuits occur when the eyes track a moving stimulus to stabilize the image on the fovea [8–10]. Finally, saccades are rapid movements of the fovea between fixation points [11]. Depending on the type of eye movement, different brain regions become activated; for example, several structures in the cerebral cortex control when and where saccades occur and cerebellar structures regulate saccade size and accuracy [12]. Furthermore, visual stimuli presented in different directions recruit specific brain regions to generate saccades. For horizontal saccades (HSs), the paramedian pontine reticular formation in the pons receives saccade initiation signals from the frontal eye field (FEF). On the other hand, the rostral interstitial nucleus of the medial longitudinal fasciculus in the midbrain receives signals from the FEF to produce vertical saccades (VSs) [12].

Saccades encompass a complex hierarchy of eye movements that includes rapid phases of nystagmus, visually guided (reflexive) saccades in response to visual stimuli and voluntary saccades [12]. Saccades are influenced by a range of cognitive and motor processes and are often characterized by their velocity, duration, accuracy and initiation time. Voluntary saccades are further divided into four types: saccades to command, predictive saccades produced in anticipation of a target, memory-guided saccades generated to the position of a previous target and antisaccades made in the opposite direction of a target [12]. Specific oculomotor tests are able to examine self-paced saccades – voluntary saccades made between two stationary targets in a fixed period of time [13]. Execution of self-paced saccades involves a pathway from the FEF and the dorsolateral prefrontal cortex to the superior colliculus[14]. The anterior cingulate cortex also plays a role in sustaining motivation to perform self-paced saccade tasks [13].

Several injuries and disorders can affect saccadic performance in humans, including Parkinson’s disease, progressive supranuclear palsy and TBI [15]. Individuals diagnosed with TBI have displayed abnormal antisaccades, memory-guided saccades and self-paced saccades, and these impairments reflect the degree of head trauma [2,15]. The severity of a TBI can be categorized as mild, moderate or severe based on a patient’s CT structural imaging scans, their level of consciousness and the duration of their post-traumatic amnesia [16]. The Glasgow coma score (GCS) is widely used and evaluates a patient’s level of consciousness using a 3–15 point scale that rates a patient’s best motor response (6 points), best verbal response (5 points) and eye opening ability (4 points), with higher scores indicating a higher state of consciousness [17]. Mild TBI (mTBI) is defined by a GCS between 13 and 15, moderate between 9 and 12, and severe between 3 and 8 [2]. mTBI is the most common form of TBI and includes concussions or brain injuries from blows to the head or body that induce neurological symptoms [2,18]. mTBI is further classified by normal CT scans, a level of consciousness of <30 min and a post-traumatic amnesia of <1 day [16].

Common tests for TBI include symptom evaluations and neuropsychological testing used for injury management [19]. While quick and convenient, symptom reports are subjective, vulnerable to under-reporting and may have limited long-term value since cognitive and visual deficits can endure after symptoms diminish [19]. Given the limitations of the common screening tools, it is important to develop and implement more objective and specific methods to assist in concussion and TBI diagnostic decision making. To this end, eye-tracking technology quickly delivers precise, objective eye movement recordings by surveying the eye several times per second [8]. With rapid advances in technology and continuing decreases in prices, sophisticated eye-tracking systems will be easily implemented in clinical settings and could serve as a valuable diagnostic tool [4]. Specifically, assessing oculomotor performance as a proxy for neural deficits caused by TBI with oculomotor assessments produces quantifiable data to complement existing TBI screening methods [16,20]. As both planning and rapidly producing saccades to targets in the visual field recruit frontoparietal areas vulnerable to damage from TBI, saccadic deficits are important to examine with eye-tracking metrics [18,21]. Common metrics for HSs include the number of fixations made during a saccade task (fixation number [#]), the ratio of saccadic velocity to accuracy (S/A ratio) and targeting metrics that measure accuracy. Accuracy metrics, such as saccade gain, measure how close eye gazes are to target stimuli by calculating the ratio of saccade amplitude to target amplitude. Position and direction errors are further indicated by the amount that saccades over or undershoot a target [22]. VS metrics also include efficiency, which evaluates how well subjects follow the vertical path between target stimuli.

Several studies have revealed deficits on saccade tasks in patients with TBI or postconcussion syndrome (PCS), with the majority focusing on measurements of HSs. These deficiencies, moreover, may reveal lingering consequences of even mTBI that traditional neuropsychological evaluations fail to identify [23]. Observed abnormalities include prolonged latencies and directional errors on memory-guided and antisaccade tasks in mTBI patients [5,14,24–27] and impaired antisaccades, memory-guided saccades and self-paced saccades in PCS patients [28] to name a few. In particular, several studies have revealed that TBI patients display impaired self-paced saccades compared with healthy controls [5,14,29]. Williams *et al.* and Mulhall *et al.* recorded the number of re-fixations within 30 s to examine the rate of self-paced saccades and found that TBI patients performed fewer saccades [14,29]. Heitger *et al*., moreover, conducted two studies of saccadic behavior following head injury and found that mTBI and PCS patients performed fewer self-paced saccades with longer intersaccadic intervals, suggesting impaired prefrontal function [25,28]. In the latter study, PCS patients also exhibited saccades with a lower peak velocity [28]. In addition, Taghdiri *et al.* observed a lower number of self-paced saccades in PCS patients, especially in patients with more recent concussions [13]. The number of saccades negatively correlated with the number of self-reported concussion symptoms and positively correlated with the fractional anisotropy value of two white matter tracts is often damaged after concussion [13]. In contrast, Phillipou *et al.* found no decrease in the rates of self-paced saccades in children with mTBI, possibly because of the small sample size used and the mild nature of patient injuries [30].

Although few studies have specifically tested vertical saccadic performance following head trauma – and none have examined vertical self-paced saccades – there is some evidence of unusual VSs in TBI patients. For example, significant differences were found between a group of mTBI patients and controls on a number of vertical saccadic parameters, including greater position errors, smaller amplitudes and smaller peak velocities and accelerations detected in the mTBI subjects [22]. Also, results from vestibular/ocular motor screening (VOMS) assessments of patients with sports-related concussion indicated that VS tests positively correlated with the total symptom score on the Post-Concussion Symptom Scale [31]. Another study using VOMS found an association between prolonged recovery time from sports-related concussion and impaired VSs [32].

Additional studies with larger sample sizes are necessary to increase the reliability and precision of results [13,23,24]. Few studies of oculomotor impairments following TBI have concentrated on self-paced saccades and even fewer have related specific deficits in self-paced saccades to TBI symptoms. The existing literature on TBI especially lacks studies investigating vertical self-paced saccades. As horizontal and VSs recruit different brain regions, further investigation is necessary to better understand potential deficits in saccadic behavior caused by TBI. Due to the limited research assessing self-paced saccade performance after TBI, additional research is needed. The purpose of this study, therefore, was to explore differences in horizontal and VSs – measured by fixation (#), S/A ratio, targeting and efficiency – between people without a history of TBI and patients clinically identified as having TBI (either mild, moderate or severe).

## Method

### Participants

A total of 195 participants were recruited to take part in the study from eye health clinics in the USA. All participants had to perform an HS test and a VS test. The participants who took part in the study were between the ages of 10 and 79 years (mean = 37.75, standard deviation = 16.49); 100 were males (51.29%) and 95 were females (48.71%). Of the 195 participants, 70.65% were white, 5.43% were Hispanic, 5.43% were Asians, 4.35% were black and 14.14% opted not to report ethnicity. Among these participants there were 64 mild TBI, 57 moderate TBI and 23 severe TBI cases that were clinically verified by a Board Certified Neurologist or Neuro-Optometrist according to medical diagnosis guidelines. There were 51 participants reporting no history of TBI. The participants with TBI had sustained their head injuries within the last 30 days prior to testing. The groups were matched by age (Table 1).

**Table 1.** Demographic data by age and gender.

Group (n)	Mean age (SD)	Females
Mild (51)	41.68 (18.57)	34
Moderate (64)	39.31 (19.37)	28
Normal (57)	38.30 (18.71)	31
Severe (23)	43.33 (16.50)	7

SD: Standard deviation.

### Apparatus

Stimuli were presented using the RightEye tests [33] on NVIDIA 24-inch 3D vision monitor fitted with an SensorMotoric Instruments (SMI) 12″ 120 Hz remote eye tracker connected to an Alienware gaming system, and a Logitech (model Y-R0017) wireless keyboard and mouse. The participants were seated in a stationary (nonwheeled) chair that could not be adjusted in height. They sat in front of a desk in a quiet, private testing room. Participants’ heads were unconstrained. The accuracy of the SMI eye tracker was 0.4° within the desired headbox of 32 cm × 21 cm at 60 cm from the screen. For standardization of testing, participants were asked to sit in front of the eye tracking system at an exact measured distance of 60 cm (ideal positioning within the headbox range of the eye tracker).

### Oculomotor tasks

Two RightEye oculomotor tests are described below.

#### HS test

In the HS test, participants were asked to look at a countdown of three, two, one in the center of the screen before moving their eyes back and forth between two dots. Their goal was to ‘target each dot’ on the left and right of the screen as quickly and accurately as possible. The targets were 10 cm apart and 1 cm in diameter. The tests were taken with a black background with white dots and lasted 10 s. The HS test provides measures of fixation percentages, fixation (#), saccade percentages and target accuracy.

#### VS test

The protocol for the VS test was the same as that for the HS test. However, the VS test was in a vertical plane.

### Procedure

Participants were recruited through advertisements placed on the internet, social media, bulletin boards and spread by word of mouth. The study was conducted in accordance with the tenets of the Declaration of Helsinki. The study protocols were approved by the Institutional Review Board of East Carolina University. The nature of the study was explained to the participants and all participants provided written consent to participate. Following informed consent, participants were asked to complete a prescreening questionnaire and an acuity vision screening where they were required to identify four shapes at 4 mm in diameter. If any of the prescreening questions were answered positively and any of the vision screening shapes were not correctly identified, then the participant was excluded from the study. Participants were excluded from the study if they reported any of the following conditions, which may have prevented successful test calibration during the prescreening process: this included vision-related issues such as extreme tropias, phorias, static visual acuity of >20/400, nystagmus, cataracts or eyelash impediments [34–38] or if they had consumed drugs or alcohol within 24 h of testing. Participants were also excluded if they were unable to pass a nine-point calibration sequence. Less than 1% of the participants fell into these categories.

Qualified participants who successfully passed the nine-point calibration sequence completed the eye tracking tests. The calibration sequence required participants to fixate one at a time on nine points displayed on the screen. The participants had to successfully fixate on at least eight out of nine points on the screen to pass the calibration sequence. Written instructions on screen and animations were provided before each test to demonstrate appropriate behavior required in each of the tests.

### Data analysis

The differences in the groups (no-TBI, mild, moderate, and severe TBI) were analyzed on clinically verified data. The comparison was evaluated using one-way univariate analysis of variance (ANOVA) on the fixation (#), S/A ratio and targeting metrics. Fixation (#) was defined as the number of gaze fixations that occurred during the total duration of the saccade task. Targeting was included as a measurement of accuracy, or how close a participant’s eye gaze was to the target stimuli while performing a saccade, while S/A ratio was defined as the ratio of speed (saccadic velocity) over accuracy. A *posthoc* analysis was conducted using Tukey’s HSD (honest significant difference) test to determine the mean differences and statistical significance between each group. The α-level was set at p < 0.05 for all statistical tests. In addition, receiving operating characteristic (ROC), area under the curve, and sensitivity and specificity were calculated for a logistic regression to predict ‘no-TBI’ versus ‘all categories of TBI’ (mild, moderate and severe). We evaluated the data using an exploratory data analysis. In this process we plotted the dataset; examined the underlying data structure; examined the data for outliers and anomalies; tested underlying assumptions; and determined optimal factor settings. In this process, we examined the data for outlier values that are outside of the areas of the distribution (at least three standard deviations) and found none that were of concern.

## Results

### Group comparison

To ensure equal groups, we compared the groups by age and gender. Using an ANOVA, we compared groups by age and revealed no difference between groups (*F* [3, 191] = 0.377; p = 0.796, ω^2^ = 0*.*001). In addition, we ran chi-squared analysis comparing groups by gender. Overall, the gender of participants did not differ by group, X^2^ (1, N = 195) = 6.457, p = 0.0954. Although the variables of horizontal and VSs can differ by age and gender, it was determined that these groups were equal and therefore age and gender is not included as variable in further analyses (Table 1).

### Horizontal saccades

**Figure 1.**
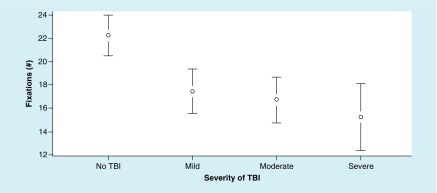
Mean values of fixation (#) at each level of traumatic brain injury severity, with 95% CI. For fixation (#) metrics – a higher value is better. TBI: Traumatic brain injury.

The ANOVA for fixation (#) metrics revealed significant differences between the groups. Fixation (#) metrics resulted in a significant main effect, F (3, 191) = 5.44; p = 0.004, ω^2^ = 0.11. In addition, Tukey’s HSD test demonstrated significant differences between the moderate and severe group and the no-TBI group; however, there was no significant difference between the mild and TBI groups (Figure 1).

**Figure 2.**
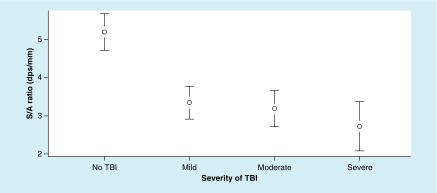
Mean values of saccadic velocity to accuracy ratio at each level of traumatic brain injury severity, with 95% CI. For saccadic velocity to accuracy ratio metrics – a higher value is better. dps: Degrees per second.

The ANOVA for S/A ratio metrics revealed significant differences between the groups, F (3, 191) = 12.38; p = < 0.0001, ω^2^ = 0.25. In addition, Tukey’s HSD test demonstrated significant differences between each TBI group and the no-TBI group (Figure 2).

**Figure 3.**
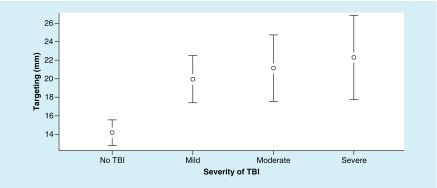
Mean values of targeting (mm) at each level of traumatic brain injury severity, with 95% CI. For targeting metrics – a lower value is better. TBI: Traumatic brain injury.

The ANOVA for targeting metrics revealed significant differences between the groups, and targeting metrics resulted in a main effect, F (3, 191) = 6.31; p = 0.015, ω^2^ = 0.08. However, Tukey’s HSD test demonstrated significant differences between TBI groups and the no-TBI group (Figure 3).

**Figure 4.**
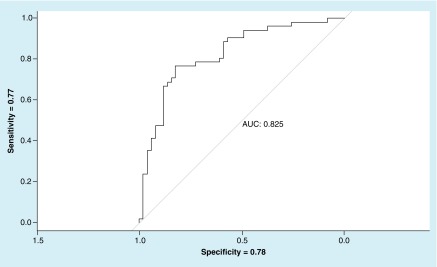
Receiving operating characteristic for horizontal saccades (fixation # and saccadic velocity to accuracy ratio) – no-traumatic brain injury versus traumatic brain injury. AUC: Area under the curve.

The logistic regression model for fixation # and S/A ratio revealed differentiated no-TBI and TBI groups. The resulting ROC curve produced an AUC value of 0.825 with sensitivity = 0.77 and specificity = 0.78 (Figure 4).

### Vertical saccades

**Figure 5.**
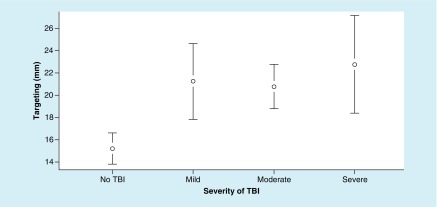
Mean values of targeting (mm) at each level of traumatic brain injury severity, with 95% CI. For targeting metrics – a lower value is better. TBI: Traumatic brain injury.

The ANOVA for targeting metrics revealed significant differences between the groups, and targeting metrics resulted in a significant main effect, F (3, 191) = 4.91; p = 0.003, ω^2^ = 0.06 (Figure 1). In addition, the Tukey’s HSD demonstrated a significant difference between each TBI group and the no-TBI group (Figure 5).

**Figure 6.**
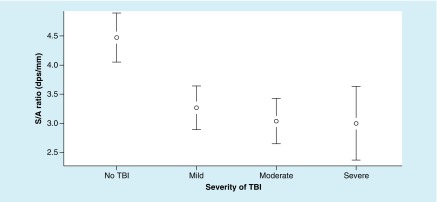
Mean values of saccadic velocity to accuracy ratio at each level of traumatic brain injury severity, with 95% CI. For saccadic velocity to accuracy ratio metrics – a higher value is better. dps: Degrees per second; S/A: The ratio of saccadic velocity to accuracy; TBI: Traumatic brain injury.

The ANOVA for S/A ratio metrics revealed significant differences between the groups, and S/A ratio metrics resulted in a significant main effect, F (3, 191) = 10.98; p < 0.0001; ω^2^ = 0.13. In addition, the Tukey’s HSD demonstrated a significant difference between each TBI group and the no-TBI group (Figure 6).

**Figure 7.**
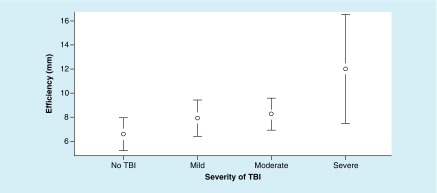
Mean values of efficiency (mm) at each level of traumatic brain injury severity, with 95% CI. For efficiency metrics – a lower value is better. TBI: Traumatic brain injury.

The ANOVA for efficiency metrics did not reveal significant differences between the groups, F (3, 191) = 0.2.34; p = 0.074, ω^2^ = 0.020 (Figure 7).

**Figure 8.**
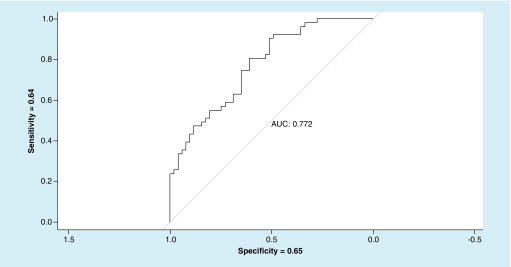
Receiving operating characteristic for vertical saccades (targeting and saccadic velocity to accuracy ratio) – no-traumatic brain injury versus traumatic brain injury. AUC: Area under the curve.

The logistic regression model was built with two metrics, targeting and S/A ratio, which came out as significant in a-priori and post-hoc analysis. Resulting ROC curve produced an area under the curve (AUC) value of 0.772 with sensitivity = 0.64 and specificity = 0.65 (Figure 8).

## Discussion

This is the first study to systematically examine the use of eye-tracking technology to study differences in HS and VS between people with no history of TBI and patients with a clinical diagnosis of TBI. Given that planning and rapidly producing saccades to targets in the visual field recruit frontoparietal areas vulnerable to damage from TBI, saccadic deficits are important to examine with eye-tracking metrics [18,21]. The technology has therefore been developed to provide an indication of neural dysfunction via the measurement of eye movements. The tests in the present study measured fixation (#), the ratio of S/A ratio and targeting metrics alongside efficiency for VSs to quantify the accuracy of the individual’s eye movements. For horizontal saccades, the S/A ratio metric was the most sensitive, showing significant differences between each group and the ratio of saccadic velocity to accuracy. Analysis of the fixation (#) suggested that the tests were able to differentiate between individuals with severe and moderate TBI and those with mild TBI and no-TBI reported. Furthermore, the analysis of the targeting metrics suggests that eye tracking can distinguish between individuals reporting a TBI and those unaffected. Overall, the sensitivity and specificity values for the horizontal saccade metrics are promising in differentiating TBI cases from no-TBI, considering this analysis only accounts for two metrics. Similarly, for the VSs test, the individual metrics were able to detect differences in individuals reporting a TBI and those who did not in both S/A ratio and targeting metrics. The efficiency metric on its own failed to discern any differences between groups, although a main effect was present. Overall, the sensitivity and specificity values for the VS test metrics are promising in differentiating TBI cases from no-TBI, considering this analysis only accounts for two metrics.

As the eye-tracking tests at the center of this study measure a combination of useful metrics, it is suggested that as a whole this is a useful adjunct to the more established diagnostic tools for TBI symptom detection. While quick and convenient, these commonly used symptom reports and neuropsychological tests are subjective, susceptible to under-reporting and may have minimal long-term use [19]. The standard test for concussion in sport, for example, is the third version of the Sport Concussion Assessment Tool (SCAT-3), which involves a physical exam, a GCS score, and cognitive and sensorimotor evaluations [39]. This testing battery includes other tests, such as the standardized assessment of concussion (SAC) and the balance error scoring system (BESS); however, none of these specifically test vision [31]. The defense automated neurobehavioral assessment (DANA) is a new mobile phone application that consists of cognitive and psychological tests to rapidly and reliably identify cases of TBI [40], that being said, neuropsychological tests such as the DANA have the potential for patient motivation to affect results [22,41]. While the King–Devick test (KDT) more commonly measures reading speed and language production, it also includes an evaluation of saccades; however, this test does not examine other eye movements often impaired after TBI [31,39,42]. VOMS provides a more complete assessment of eye movements to help clinicians evaluate vestibular and ocular abnormalities after concussion [43]. The VOMS tests saccades, smooth pursuits, fixations, convergence and the vestibular–ocular reflex [19]. Although it can distinguish concussed athletes from healthy controls, the VOMS relies on subjective reports of symptoms that may introduce error from recall bias and under-reporting. Also, VOMS cannot detect specificity beyond gross eye movement observation by the clinician [31].

Certainly, there is thus great benefit in testing individuals with suspected TBIs with these eye-tracking tests to confirm (or deny) the presence of TBIs given that this is the only method of objectively and accurately recording and analyzing visual behavior. The objectivity and accuracy of eye tracking addresses many of the criticisms of the existing TBI symptom detection methods and therefore addresses problems with subjectivity, under- and over-reporting as well as issues with patient motivation. There may also be merit in using such tests to measure progress in rehabilitation from TBI. Given that these tests are simple and quick to administer, investigations into the use in sport settings may prove fruitful, especially for the purposes of ‘return-to-play’ decisions. However, given the infancy in the use of eye tracking as a means of symptom detection in those with TBIs, it is recommended that further research be conducted to amass a broader sample upon which to compare these results to. Although the sample size (n = 287) of the present study is relatively small, it was considered adequate though a broader sample of participants from a wider geographical area is needed to ensure enough international diversity upon which to compare results.

## Conclusion

TBI is a significant international public health concern, yet the methods currently used for its detection are inconsistent and subject to limitations. This study has demonstrated that specialized eye-tracking tests are a useful precise and objective measure of underlying neurological health that indicates the presence of a TBI. Furthermore, particular metrics have shown to be able to differentiate in the level of severity of the TBI. In conclusion, it is suggested that testing HS and VS using eye-tracking technology is a useful adjunct to the more established tests and protocols currently used by clinicians.

## Future perspective

It is clear that our knowledge of TBI is continually evolving and with this comes an understanding of how to harness new technologies to probe areas of the brain implicated in TBI to improve its diagnosis and treatment. Over the next 5–10 years it is predicted that the use of simple, user-friendly eye-tracking tests such as those analyzed in the present study will become commonplace in the diagnosis of TBI, its severity and in the monitoring of recovery from this condition.

**Executive summary**

- Traumatic brain injury (TBI) is one of the most concerning socio-economic health issues in today’s society.
- Although there are a number of tests and protocols to detect symptoms of TBI, limitations inherent to these tests warrant caution in interpreting the validity of their diagnostic use and the objectivity of the data provided.
- Oculomotor behavior is a promising neuropsychological endophenotype, as it reflects abnormalities of complex neurocircuitry in an objective examination of neurological health.
- Several studies have revealed deficits on saccade tasks in patients with TBI or postconcussion syndrome.
- Eye-tracking technology quickly delivers precise, objective eye movement recordings by surveying the eye several times per second.
- This technology can be used to measure horizontal and vertical saccades via a number of discrete metrics.
- The present study shows that using eye-tracking technology to measure horizontal and vertical saccades is a simple, quick and accurate measure that is able to accurately differentiate between individuals with different levels of severity of TBI and those who have not sustained a TBI.
